# Predicting Inpatient Detoxification Outcome of Alcohol and Drug Dependent Patients: The Influence of Sociodemographic Environment, Motivation, Impulsivity, and Medical Comorbidities

**DOI:** 10.1155/2017/6415831

**Published:** 2017-03-06

**Authors:** Yvonne Sofin, Heidi Danker-Hopfe, Tina Gooren, Peter Neu

**Affiliations:** ^1^Jewish Hospital Berlin, Clinic for Psychiatry and Psychotherapy, Heinz-Galinski-Str. 1, 13347 Berlin, Germany; ^2^Competence Center for Sleep Medicine, Charité-School of Medicine, Campus Benjamin Franklin, Hindenburgdamm 30, 12203 Berlin, Germany; ^3^Charité-School of Medicine, Campus Benjamin Franklin, Hindenburgdamm 30, 12203 Berlin, Germany

## Abstract

*Aims*. This prospective study aims to identify patient characteristics as predictors for treatment outcome during inpatient detoxification treatment for drug and alcohol dependent patients.* Methods*. A mixed gender sample of 832 consecutively admitted drug and alcohol dependent patients were interviewed by an experienced physician. The impact of a variety of factors concerning social environment, therapy motivation, impulsivity related variables, medical history, and addiction severity on treatment outcome was examined.* Results*. 525 (63.1%) of the patients completed detoxification treatment whereas 307 (36.9%) dropped out prematurely. Being female, living in a partnership, having children, being employed, and having good education were predictive for a positive outcome. Family, health, the fear of losing the job, prosecution, and emergency admission were significant motivational predictors for treatment outcome. Being younger, history of imprisonment, and the number of previous drop-outs were predictive for a negative outcome.* Conclusions*. Variables concerning social environment and the number of previous drop-outs have been identified as best predictors for treatment outcome. Socially stable patients benefit from the current treatment setting and treatment shall be adapted for patients with negative predictors. Treatment may consequently be tailored with respect to intervention type, duration, and intensity to improve the outcome for those patients that fulfil criteria with negative impact on treatment retention.

## 1. Introduction

Addiction is a chronic disease that affects millions of individuals worldwide. In Germany, alcohol dependence is the most serious, expensive, and socially disruptive health issue [1]. In industrialized countries, alcoholism is among the leading causes of death [2]. Qualified detoxification treatment (QDT) is the first step in inpatient substance abuse treatment. Premature discontinuation of QDT is a serious and common complication in the detoxification treatment [3]. The risk of relapse substantially determines prognosis and mortality of the disease [4]. Treatment noncompletion is generally associated with poor success and an unfavourable long-term outcome [5]. Regular completion of treatment is therefore a key success criterion of inpatient detoxification treatment as the discontinuation of therapy is usually accompanied by a relapse [6]. For both, alcohol dependent and drug dependent patients, high drop-out rates were reported in literature. Thus, a drop-out rate of 33% has been reported for alcohol dependents [7], while the observed drop-out rate for illicit drug users was even higher at 50% [8]. Therefore it is of high importance to identify determinants and risk factors of unplanned premature discharge and to adapt the treatment for the individual patient accordingly. Some predictors were repeatedly identified in previous studies: for drug addiction, level of education, unemployment, and delinquency were predictive for relapse [9–11]. For alcohol addiction, significant predictors were the number of prior hospitalizations for detoxification, dependence severity, and psychopathologic rating [1, 12, 13]. The link between illicit drug use and crime is well documented [14]. Backmund et al. [10] found history of imprisonment and currently being on probation to be significant predictors of completing detoxification treatment. Previous studies indicated that lower injection frequency before admission was associated with twofold increases in the likelihood of having favourable follow-up outcomes on illicit drug use, alcohol use, and criminal involvement [15].

Additionally, impulsivity plays a major role in substance use disorders [16]. Impulsiveness involves behaviour characterized by little or no forethought or consideration of the consequences [17]. Impulsive actions are therefore often poorly conceived or inappropriate to the situation and result in undesirable consequences, for example, choosing short-term gains over long-term gains [18]. Suicide attempts are often regarded as impulsive acts [19]. According to Wines Jr. et al. [20], previous suicide attempts are common in substance-dependent individuals. Nearly half of the drug dependent patients (45%) reported having attempted suicide at some point of their life [21]. Pretreatment suicide attempts are associated with a higher likelihood of relapse [22].

The purpose of the present study was to identify further predictors of premature discharge during inpatient QDT for drug and alcohol dependent patients to thereby achieve better outcomes in terms of treatment completion for drug or alcohol dependent patients. With regard to the high costs in public healthcare systems, prediction of treatment outcome provides the opportunity to identify client groups that achieve poorer outcomes and identify targets in treatment to improve inpatient detoxification treatment. Clinicians should be enabled to set realistic treatment goals and adapt intervention duration and intensity. We therefore conducted a prospective analysis to investigate the influence of sociodemographic as well as medical variables on QDT outcome on patients suffering from addiction.

## 2. Methods

### 2.1. Participants

During the year 2012, 914 consecutively drug and alcohol dependent patients admitted to the hospital were screened and asked for participation. 832 patients were included in the study. All patients fulfilled the DSM-IV criteria for substance addiction and gave their written informed consent to participate in this study. Exclusion criterion was noncapacity of giving informed consent (severe organic or psychiatric disorders like Korsakow syndrome, etc.).

### 2.2. Setting and Treatment Procedure

The study was conducted on two specialized inpatient units for qualified detoxification treatment of addiction diseases in a psychiatric hospital in Berlin, Germany. The treating team comprised medical doctors, psychologists, specialized nurses, occupational therapists, physiotherapists, and social workers. The qualified detoxification treatment enriches detoxification treatment with psychoeducation and relapse prevention. It consists of three steps. While detoxification the patients were withdrawn from the drug and, where needed, withdrawal symptoms were treated. In the second step, the patients had to attend at least ten group-therapy sessions and five psychoeducational group-sessions. In the third step, the preparation of transition to a long-term follow-up treatment after hospital discharge including the attendance of five self-help groups outside the clinic was conducted.

The average treatment took between 12 and 16 days but could last longer in case of persisting withdrawal symptoms or particularly severe general condition.

Clomethiazole at tapered doses was used for alcohol detoxification. Methadone at tapered doses was used for opioid detoxification. For cannabis, amphetamines and cocaine detoxification abrupt cessation without medical support was chosen. The severity of alcohol withdrawal symptoms was captured according to the CIWA Withdrawal Score [23].

### 2.3. Diagnostic Criteria

For diagnosis of addiction and concomitant diseases Diagnostic and Statistical Manual (DSM) edition IV was applied.

### 2.4. Definition of Outcome Criteria

The treatment was considered successfully completed if the patient remained abstinent while hospital stay and participated in the treatment program as described above until regular discharge. The attendance to at least ten group-therapy sessions, five psychoeducational group-sessions, and five self-help groups outside the clinic was mandatory.

The treatment was considered aborted if the patient left against medical advice or due to disciplinary early discharge. Substance use or refusal to participate in the treatment program led to disciplinary discharge.

### 2.5. Data Analysis

Data on the patient's social environment consisting of information on their living and domestic situation, children, graduation, employment, and native language were collected. Additionally, the patient's therapy motivation was asked upon hospital admission (Table 2). Answers are comprised of fear of losing the partner or family, harming his or her health, fear of losing the job and/or residence, making a therapy instead of imprisonment, the aim of abstinence, and other motivations. Some patients did not specify their motivation.

The patient's impulsiveness was measured by data on experience of violence, aggressive behaviour, suicidal tendency, and information on constraints in terms of judicial proceedings, probation, and imprisonment. Further, the impact of intravenous drug use and the effect of genetic predisposition on impulsiveness related behaviour expressed by addiction and suicidal tendency in relatives were elaborated.

All patients were admitted electively for qualified detoxification treatment except for emergency admissions.

Data on medical history comprised the addiction diagnoses and, if applicable, addiction associated disorders, for example, central nervous system damage.

All data were captured by an experienced physician during structured face to face admission interview. Statistical analyses were carried out using SAS (statistical analysis system) software by SAS Institute. It was separately examined for all variables, whether there was a difference between the patients with and without premature treatment completion. For nominal and ordinal scaled variables, the examination was carried out with log likelihood Chi square test. For interval scaled data, the relationship between the respective variable and premature discharge was analyzed with a *t*-test for independent samples (if normal distribution was assumed) or with a Wilcoxon 2-sample test (if normal distribution could not be assumed). Normal distribution was tested using the Kolmogorov-Smirnov test with a two-sided significance level of *p* < 0.01. Tests on group differences were examined with a two-sided significance level of *p* < 0.05. *p* values of 0.05 or less were considered statistically significant. Furthermore logistic regression analyses were performed separately for the 4 clusters of variables: (1) sociodemographic determinants, (2) motivational and addiction associated determinants, (3) impulsiveness related characteristics of patients, and (4) determinants from the patient's medical history to identify significant predictors of the treatment outcome. In the logistic regression the probability of a premature treatment completion was modelled.

## 3. Results

### 3.1. Patient Characteristics

832 patients were included in the study. 619 (74%) patients were male and 213 (26%) were female. The mean age was 44 (±13) years. Sociodemographic details of the sample are given in Table 1. The patient sample was characterized by a high number of patients without partnership (60.3%) and 45.2% participants that were dependent on welfare. 32.7% were unskilled and more than one in ten patients was homeless. Asked about their therapy motivation, health and family were frequent answers with 27.8 and 20.0%, respectively (see Table 2). Of the 832 patients included, the most frequent diagnosis was alcohol addiction with 71.4% followed by 12.3% opioid abuse. While 64.7% suffered of only one addiction, 35.3% had more than one diagnosed addiction (Table 4(a)).

### 3.2. Determinants for Premature Treatment Drop-Out

Overall, 525 (63.1%) of the 832 patients completed detoxification treatment whereas 307 (36.9%) dropped out of the program. The 307 individuals of the drop-out group comprised 249 patients (81.1%) that prematurely terminated the treatment on their own initiative while 58 (18.9%) were discharged due to disciplinary reasons. Patients in the treatment drop-out group were significantly younger (39 years) than the patients who completed the treatment (46 years) and men dropped out more often than women (38.9% versus 31.0%).

In this study, all tested sociodemographic pretreatment variables showed a significant influence on the treatment outcome. Patients that were female, lived in a partnership, or were at least together with other individuals, had children and were employed, were well-educated, and spoke German as native language were more likely to finish the treatment successfully. Having children had a positive impact on the treatment outcome. At least 65.7% of the patients with children completed the treatment regularly, whereas only 57.2% of the childless patients completed the treatment. In our study, the increasing number of children did not correlate with an increasing probability of treatment completion. The higher the patient's graduation and occupational training was, the higher the probability to complete the treatment regularly was (Table 1(a)). Logistic regression revealed that being younger and being unemployed significantly increased the risk of a unplanned, premature discharge. But it did not confirm the influence of the gender on treatment outcome (Table 1(b)).

Family, health, the fear of losing the job, prosecution, and emergency admission were significant motivational predictors for QDT outcome. For the patients that did not specify a certain motivation or named abstinence as treatment motivation, no significant influence was shown. Individuals with no prior detoxification significantly more often completed the treatment regularly. Also, we found that patients with no previous treatment drop-outs significantly more often completed QDT (*p* = 0.0001). The duration of the longest period of the patients' abstinence (*p* = 0.0874) was not predictive for an early treatment drop-out (Table 2(a)).

Logistic regression with all motivation and treatment variables revealed that treatment motivation is not significant for treatment outcome but that the number of previous drop-outs was the best predictors for outcome. Subjects with one or two previous early discharges had a 4.7-fold increased risk (95%-CI: 2.9; 7.4) and for subjects with three or more previous premature discharges the risk increased even to 10.4 (95%-CI: 4.0; 27.6). The number of previous premature drop-outs was hence the best predictor of all 4 clusters examined in our study. Logistic regression further confirmed that duration of abstinence is not predictive for treatment retention, although the longest duration of abstinence was twice as long in patients that completed the treatment as in patients that dropped out (Table 2(b)).

Table 3(a) illustrates the impact of personality in terms of impulsiveness related variables on treatment outcome. Among the impulsiveness related variables, experiences of violence, aggressive behaviour towards third parties, history of imprisonment, and intravenous drug use influenced treatment outcomes negatively. For subjects with prior suicide attempts the number of drop-outs was not statistically significant. Similarly, genetic predisposition did not predict treatment outcome, neither concerning relatives of first or second degree with addiction nor for relatives of first or second degree with suicidal behaviour.

These findings were verified by logistic regression analysis. In particular, patients without a history of imprisonment (OR: 0.50; 95%-CI: 0.33; 0.76) and patients without intravenous drug use (OR: 0.45; 95%-CI: 0.30; 0.69) have a significantly reduced risk of premature treatment completion (Table 3(b)).

Data on the patient's medical history were analyzed (Table 4(a)). The presence of an addiction related infection (*p* = 0.0145) or a central nervous system disorder (*p* = 0.0416) was predictive for treatment outcome. On the other hand, comorbid gastrointestinal disorders (*p* = 0.0554) or peripheral central nervous system damage (*p* = 0.7909) was not predictive for treatment outcome. Considering the diagnosed addiction, the first as well as the second addictive disorders were significantly related to treatment outcome whereas the third diagnosed addiction was not (*p* = 0.0865). Of 538 patients diagnosed with only one addiction, 67.3% completed the treatment successfully. In our study, patients with first addiction diagnosis of alcohol addiction or pathological gambling completed the treatment in 70% and 100%, respectively, of the cases. In contrast, more than half of the individuals with cannabis, opioid, or multiple drug abuse dropped out the treatment.

Having an additional, nonaddiction related diagnosis had a significant beneficial effect on treatment outcome (*p* = 0.0001). Logistic regression confirms that having no disorder other than the addictive disorder doubled the risk of premature treatment completion significantly (OR 2.07, 95% OR: 1.50–2.86). Furthermore, it revealed that having alcohol dependency as the first diagnosed addictive disorder increased the risk of premature treatment drop-out significantly (see Table 4(b)). Opioid dependency increased the risk to 3.23 (95% OR: 1.98–5.25), while subjects dependent from cannabis, sedatives/hypnotics, cocaine, pathological gambling, or multiple drug use have twice the risk of alcohol addicted people (OR 2.12, 95% OR: 1.40–3.21) to drop out from treatment.

## 4. Discussion

The aim of the present study was to identify predictors of premature discharge during inpatient QDT for alcohol and drug dependent patients. This study showed that drug dependent patients bare an elevated risk of premature treatment drop-out compared to alcohol dependent patients. These findings are consistent with past research described in literature. Braune et al. described drop-out rates of 43.3% for alcohol dependent and 62.4% for drug dependent patients [9]. Our results further suggest that, for patients with multiple addictions, the main addiction, as well as the second, if applicable, has an influence on treatment outcome, but not if they suffer of more than two addictions. Further investigation should be carried out to verify if there is indeed no distinction in patients using more than two substances. In our study, 100% of the pathologic gamblers succeeded, but further investigations on the influence of pathologic gambling on the likelihood of relapse shall be conducted to verify this finding, as in the present study only four patients with pathologic gambling were included which limits the generalisability of the finding.

In our study, being female was a predictor for a better treatment outcome. But the result of logistic regression analysis suggests that not the gender itself but associated attributes of the female group influenced treatment outcome. This result differs from data in other studies where the drop-out rates were equal for men and women [23] or females relapsed significantly more often [24]. In our study, however, women were higher educated and were more frequently employed compared to men. Our findings indicate that a social network is supportive for a successful detoxification treatment and are consistent with other studies [1, 25]. Patients with higher education and employment live in better economic conditions and it is likely that they have a greater social network as well as an established daily structure. It is reasonable that children increase the probability of successful QDT as the responsibility for their wellbeing is likely to have a high influence on therapy motivation. This is consistent with our finding that family was a significant motivational predictor for treatment outcome. Asked about their motivation, patients who feared prison dropped out noticeably more often than patients that mentioned other treatment motivations. This result may be influenced by the fact that delinquent patients live in unstable and unsupportive social networks. Furthermore, the motivation for their treatment was not intrinsic but forced involuntarily, as therapy was stipulated by court order to avoid imprisonment. As logistic regression did not show any significant impact of motivational variables, it seems likely that the kind of motivation is less important than having a therapy motivation at all and that again other attributes represented in those patients influenced treatment outcome.

As expected, a negative association was found for violence and aggression on the treatment outcome of our patients. Available figures indicate that 20% to 40% of all adults were exposed to domestic violence during childhood or adolescence [25]. Children from families with different parental problems such as domestic violence and mental illness are a well-known at-risk group for various mental health and social problems [26].

As found by many authors, imprisonment and intravenous drug use were highly significant predictors for treatment retention. These patient attributes indicate a certain severity of addiction that impedes the detoxification treatment. These findings suggest that staying in treatment for a longer time and segregating patients from their environmental influences could increase their level of persistence.

Patients with no previous detoxification treatment and no previous drop-outs significantly more often completed QDT. These findings were in line with the results of Wagner et al. [27] who found a strong negative impact on abstinence probability depending on the number of inpatient detoxification treatments. It can be assumed that premature drop-outs and repeated detoxification treatments weaken the patient's self-efficacy and thereby increase the inhibition threshold to seek help. Interestingly, we found a decreased drop-out risk for patients that had more than 10 detoxification treatments. These patients may be more distressed by their repeated relapses and consequently engage themselves more actively in treatment. A further possible explanation is that patients with repeated detoxification treatments gain and increase profound knowledge of their disease and its treatment during QDT.

“Duration of substance dependency” was a further treatment variable that has been evaluated in the present study and that was also found to be a predictor for treatment outcome. This observation is in agreement with previous studies [1, 28] and suggests the influence of dependency severity on QDT outcome.

Comorbid infections or central nervous system disorders were predictive for treatment outcome. The impact of increased medical severity was supported by other studies [9, 29] and is consistent with our findings. We conclude that the decreased medical condition and the curative treatment that the patients receive, respectively, may serve as an additional motivational factor for treatment retention. Additionally, these patients received an increased attention, not only from psychiatric but also from somatic doctors.

All assessments evaluated in this study, despite medical condition, were based on patient self-report. The present study does not allow corroboration of the patient's statements.

Although smoking highly contributes to the high costs in public healthcare systems, smokers wishing to quit smoking were not included in our study as smoking cessation requires different intervention types. In spite of these limitations, we were able to identify numerous variables with potential influence on successful inpatient qualified detoxification treatment.

In summary, younger age, male sex, living alone, being childless, a low level of education, no employment, history of imprisonment, intravenous drug use, being drug dependent, and in particular a high number of previous drop-outs were predictive for a premature treatment drop-out. Better social network in terms of family, employment and education, and a lower dependency severity positively predicted treatment outcome. These findings suggest that socially stable patients benefit from the current treatment setting and that treatment shall be adapted for the patients with negative predictors. Treatment may consequently be tailored with respect to intervention type, duration, and intensity to improve the outcome for those patients that fulfil criteria with negative impact on treatment retention.

## Figures and Tables

**Table 1a:** (a) Results of likelihood Chi square test for sociodemographic determinants of premature treatment drop-out.

Characteristics	Total	Treatment completed	Dropped out of treatment	*p*
	*N* = 832	*N* = 525	*N* = 307	
Age (years)	43.8	46.3	39.4	**0.0001**
Sex				**0.0365**
Male	619 (74.4%)	378 (61.1%)	241 (38.9%)	
Female	213 (25.6%)	147 (69.0%)	66 (31.0%)	
Partnership				**0.0020**
Living in a partnership	330 (39.7%)	229 (69.4%)	101 (30.6%)	
No partnership	501 (60.3%)	295 (58.9%)	206 (41.1%)	
Living situation				**0.0469**
Living alone	456 (54.8%)	274 (60.1%)	182 (39.9%)	
Living with other(s)	376 (45.2%)	251 (66.8%)	125 (33.2%)	
Children				**0.0026**
No children	418 (50.2%)	239 (57.2%)	179 (42.8%)	
One child	173 (20.8%)	124 (71.7%)	49 (28.3%)	
Two children	166 (20.0%)	109 (65.7%)	57 (34.3%)	
Three or more children	75 (9.0%)	53 (70.7%)	22 (29.3%)	
Graduation				**0.0001**
High school (13 years of school)	158 (19.0%)	116 (73.4%)	42 (26.6%)	
Realschule (10 years of school)	235 (28.3%)	170 (72.3%)	65 (27.7%)	
Hauptschule (9 years of school)	363 (43.6%)	205 (56.5%)	158 (43.5%)	
No graduation	76 (9.1%)	34 (44.7%)	42 (55.3%)	
Occupational training				**0.0001**
Academic studies	86 (10.4%)	69 (80.2%)	17 (19.8%)	
Apprenticeship	473 (56.9%)	324 (68.5%)	149 (31.5%)	
Unskilled	272 (32.7%)	132 (48.5%)	140 (51.5%)	
Employment				**0.0001**
Employed	226 (27.3%)	174 (77.0%)	52 (23.0%)	
Pensioned	104 (12.6%)	82 (78.9%)	22 (21.1%)	
Welfare	375 (45.2%)	194 (51.7%)	181 (48.3%)	
Unemployed	124 (14.9%)	73 (58.9%)	51 (41.1%)	
Residence				**0.0001**
Living in own residence	599 (72.0%)	396 (66.1%)	203 (33.9%)	
Assisted living	81 (9.7%)	55 (67.9%)	26 (32.1%)	
Other	50 (6.0%)	17 (34.0%)	33 (66.0%)	
Homeless	102 (12.3%)	57 (55.9%)	45 (44.1%)	
Mother tongue				**0.0041**
German	646 (77.1%)	426 (65.9%)	220 (34.1%)	
Foreign mother tongue	181 (21.9%)	96 (53.0%)	85 (47.0%)	

**Table 1b:** (b) Results of logistic regression analysis with sociodemographic determinants of premature treatment drop-out.

Characteristic	OR	95% CI	Wald Chi2	*p*
Age	**0.96**	**0.95**–**0.98**	**19.32**	**<0.0001**
Sex				
Male	1.00			
Female	0.95	0.66–1.37	0.08	0.7789
Partnership				
Living in a partnership	1.00			
No partnership	1.02	0.64–1.63	0.01	0.9405
Living situation				
Living alone	1.00			
Living with other(s)	0.76	0.48–1.22	1.23	0.2572
Children				
No children	1.00			
One child	0.72	0.47–1.11	2.25	0.1336
Two children	1.17	0.75–1.81	2.21	0.1371
Three or more children	0.85	0.46–1.55	0.14	0.7090
Graduation				
High school (13 years of school)	1.00			
Realschule (10 years of school)	0.86	0.48–1.57	3.66	0.0556
Hauptschule (9 years of school)	1.47	0.83–2.59	2.92	0.0877
No graduation	1.47	0.70–3.12	1.06	0.3036
Occupational training				
Academic studies	1.00			
Apprenticeship	1.13	0.53–2.44	0.06	0.8065
Unskilled	1.44	0.63–3.27	1.31	0.2533
Employment				
Employed	1.00			
Pensioned	1.41	0.74–2.69	0.01	0.9503
Welfare	1.52	0.91–2.53	0.10	0.7461
Unemployed	**1.97**	**1.30**–**2.98**	**6.00**	**0.0143**
Residence				
Living in own residence	1.00			
Assisted living	0.70	0.41–1.20	3.70	0.0543
Other	1.67	0.80–3.47	2.54	0.1109
Homeless	1.10	0.69–1.76	0.03	0.8670
Mother tongue				
German	1.00			
Foreign mother tongue	1.35	0.93–1.97	2.46	0.1170

**Table 2a:** (a) Results of likelihood Chi square test for motivational and addiction associated determinants of premature treatment drop-out.

Characteristics	Total	Treatment completed	Dropped out of treatment	*p*
	*N* = 832	*N* = 525	*N* = 307	
Motivation^*∗∗*^				
Partner/family	166 (20.0%)	119 (71.7%)	47 (28.3%)	**0.0093**
Health	231 (27.8%)	164 (71.0%)	67 (29.0%)	**0.0031**
Job/residence	148 (17.8%)	104 (70.3%)	44 (29.7%)	**0.0435**
Prison (therapy instead of penalty)	15 (1.8%)	5 (33.3%)	10 (66.7%)	**0.0184**
Abstinence	164 (19.7%)	100 (61.0%)	64 (39.0%)	0.5302
Emergency	69 (8.0%)	35 (50.7%)	34 (49.3%)	**0.0285**
Other	221 (26.6%)	134 (60.6%)	87 (39.4%)	0.3766
Motivation not specified	41 (4.9%)	21 (51.2%)	20 (48.8%)	0.1115
Number of previous detoxification treatments				**0.0002**
None	295 (35.5%)	209 (70.9%)	86 (29.1%)	
1-2 detoxification treatments	269 (32.3%)	173 (64.3%)	96 (35.7%)	
3–10 detoxification treatments	210 (25.2%)	110 (52.4%)	100 (47.6%)	
11–20 detoxification treatments	39 (4.7%)	25 (64.1%)	14 (35.9%)	
More than 20 detoxification treatments	19 (2.3%)	8 (42.1%)	11 (57.9%)	
Duration of substance dependency (years)	15.2	15.6	14.4	0.1139
Longest period of abstinence (months)	12.7	15	8.8	0.0874
Number of previous drop-outs				**0.0001**
None	639 (76.8%)	457 (71.5%)	182 (28.5%)	
1-2 drop-outs	149 (17.9%)	54 (36.2%)	95 (63.8%)	
3 or more drop-outs	44 (5.3%)	14 (31.8%)	30 (68.2%)	

^*∗∗*^Multiple selections were allowed.

**Table 2b:** (b) Results of logistic regression analysis with motivational and treatment history variables as determinants.

Characteristic	OR	95% CI	Wald Chi2	*p*
Motivation				
Partner/family (no versus yes)	1.41	0.90–2.20	2.20	0.1376
Health (no versus yes)	1.42	0.91–2.24	2.43	0.1191
Job/residence (no versus yes)	1.45	0.90–2.32	2.35	0.1255
Prison (therapy instead of penalty)	0.42	0.13–1.42	1.95	0.1628
Abstinence (no versus yes)	0.97	0.59–1.58	0.02	0.8964
Emergency (no versus yes)	0.81	0.42–1.58	0.38	0.5389
Other (no versus yes)	0.95	0.60–1.52	0.04	0.8352
Not specified (no versus yes)	0.89	0.39–2.00	0.08	0.7724
Number of previous detoxification treatments				
None	1.00			
1-2 detoxification treatments	1.15	0.77–1.71	3.39	0.0654
3–10 detoxification treatments	1.08	0.65–1.81	2.77	0.0959
11–20 detoxification treatments	**0.36**	**0.14–0.93**	**5.12**	**0.0237**
More than 20 detoxification treatments	0.59	0.16–2.20	0.29	0.5926
Duration of substance dependency (years)	**0.97**	**0.96–0.99**	**7.05**	**0.0079**
Longest period of abstinence (months)	0.99	0.99–1.00	3.65	0.0560
Number of previous drop-outs				
None	1.00			
1-2 drop-outs	**4.67**	**2.93–7.45**	1.64	0.2001
3 or more drop-outs	**10.45**	**3.97–27.56**	**11.14**	**0.0008**

**Table 3a:** (a) Results of likelihood Chi square test for impulsiveness related variables associated determinants of premature treatment drop-out.

Characteristics	Total	Treatment completed	Dropped out of treatment	*p*
	*N* = 832	*N* = 525	*N* = 307	
Patient experienced abuse or violence				**0.0475**
Yes	192 (25.7%)	110 (57.3%)	82 (42.7%)	
No	554 (74.3%)	362 (65.3%)	192 (34.7%)	
Documented cases of aggressive behavior towards others				**0.0082**
Yes	141 (16.9%)	75 (53.2%)	66 (46.8%)	
No	691 (83.1%)	450 (65.1%)	241 (34.9%)	
Suicide attempts				0.5113
None	704 (84.9%)	448 (63.6%)	256 (36.4%)	
1 attempt	78 (9.4%)	47 (60.3%)	31 (39.7%)	
2 attempts	28 (3.4%)	18 (64.3%)	10 (35.7%)	
3 or more attempts	19 (2.3%)	9 (47.4%)	10 (52.6%)	
Patients in judicial proceeding				0.8027
Yes	15 (1.8%)	9 (60.0%)	6 (40.0%)	
No	817 (98.2%)	516 (63.2%)	301 (36.8%)	
Patients on probation				0.0813
Yes	33 (4.0%)	16 (48.5%)	17 (51.5%)	
No	799 (96.0%)	509 (63.7%)	290 (36.3%)	
Patients with history of imprisonment				**0.0001**
Yes	700 (84.1%)	58 (43.9%)	74 (56.1%)	
No	132 (15.9%)	467 (66.7%)	233 (33.3%)	
Intravenous drug use				**0.0001**
Yes	117 (14.1%)	49 (41.9%)	68 (58.1%)	
No	715 (85.9%)	476 (66.6%)	239 (33.4%)	
Number of relatives of first degree with addiction disorder				0.9800
None	490 (59.0%)	311 (63.5%)	179 (36.5%)	
1	260 (31.3%)	164 (63.1%)	96 (36.9%)	
2	64 (7.7%)	38 (59.4%)	26 (40.6%)	
3	14 (1.7%)	9 (64.3%)	5 (35.7%)	
4	3 (0.3%)	2 (66.7%)	1 (33.3%)	
Addiction in relatives of second degree				0.2120
Yes	207 (24.9%)	138 (66.7%)	69 (33.3%)	
No	624 (75.1%)	386 (61.9%)	238 (38.1%)	
Number of relatives of first degree with suicide				0.1211
None	809 (97.5%)	510 (63.0%)	299 (37.0%)	
1	19 (2.3%)	13 (68.4%)	6 (31.6%)	
2	2 (0.2%)	0 (0%)	2 (100%)	
Suicide attempts in relatives of second degree				0.7789
Yes	29 (3.5%)	19 (65.5%)	10 (34.5%)	
No	802 (96.5%)	505 (63.0%)	297 (37.0%)	

**Table 3b:** (b) Results of logistic regression analysis with impulsiveness related patient characteristics.

Characteristic	OR	95% CI	Wald Chi2	*p*
Patient experienced abuse or violence				
Yes	1.00			
No	0.82	0.61–1.12	1.57	0.2100
Documented cases of aggressive behaviour towards others				
Yes	1.00			
No	0.83	0.55–1.26	0.76	0.3819
Suicide attempts				
None	1.00			
1 attempt	1.03	0.62–1.70	0.01	0.9333
2 attempts	0.83	0.36–1.90	0.49	0.4832
3 or more attempts	1.43	0.54–3.81	0.63	0.4281
Patients in judicial proceeding				
Yes	1.00			
No	0.98	0.33–2.88	0.01	0.9654
Patients on probation				
Yes	1.00			
No	0.91	0.43–1.91	0.07	0.7973
Patients with history of imprisonment				
Yes	1.00			
No	**0.50**	**0.33–0.76**	**10.23**	**0.0014**
Intravenous drug use				
Yes	1.00			
No	**0.45**	**0.30–0.69**	**13.37**	**0.0003**
Number of first-degree relatives with addiction disorder				
Yes	1.00			
No	1.00	0.74–1.36	0.01	0.9929
Addiction in relatives of second degree				
Yes	1.00			
No	1.19	0.84–1.69	0.98	0.3232
Number of first-degree relatives with suicide				
Yes	1.00			
No	1.13	0.43–2.98	0.06	0.8018
Suicide attempts in relatives of second degree				
Yes	1.00			
No	1.12	0.50–2.53	0.08	0.7809

**Table 4a:** (a) Results of likelihood Chi square test for medical determinants of premature treatment drop-out.

Characteristics	Total	Treatment completed	Dropped out of treatment	*p*
	*N* = 832	*N* = 525	*N* = 307	
Addiction related comorbidity				
Infection	69 (8.3%)	34 (49.3%)	35 (50.7%)	**0.0145**
Gastrointestinal disorder	289 (34.7%)	195 (67.5%)	94 (32.5%)	0.0554
CNS disorder	60 (7.2%)	45 (75.0%)	15 (25.0%)	**0.0416**
Peripheral nervous system disorder	28 (3.3%)	17 (60.7%)	11 (39.3%)	0.7909
First diagnosed addictive disorder				**0.0001**
Alcohol	594 (71.4%)	417 (70.2%)	177 (29.8%)	
Opioid	102 (12.3%)	40 (39.2%)	62 (60.8%)	
Cannabis	50 (6.0%)	22 (44.0%	28 (56.0%)	
Sedatives/hypnotics	33 (4.0%)	23 (69.7%)	10 (30.3%)	
Cocaine	13 (1.6%)	9 (69.2%)	4 (30.8%)	
Multiple drug use	36 (4.3%)	10 (27.8%)	26 (72.2%)	
Pathological gambling	4 (0.5%)	4 (100%)	0 (0%)	
Second diagnosed addictive disorder				**0.0045**
Patients with no second addiction diagnosis	538 (64.7%)	362 (67.3%)	176 (32.7%)	
Alcohol	26 (3.1%)	20 (76.9%)	6 (23.1%)	
Opioid	33 (3.9%)	14 (42.4%)	19 (57.6%)	
Opioid substitution	5 (0.6%)	2 (40%)	3 (60%)	
Cannabis	80 (9.6%)	49 (61.3%)	31 (38.7%)	
Sedatives/hypnotics	40 (4.8%)	24 (60.0%)	16 (40.0%)	
Cocaine	30 (3.6%)	14 (46.7%)	16 (53.3%)	
Stimulants	9 (1.0%)	5 (55.6%)	4 (44.4%)	
Multiple drug use	67 (8.0%)	32 (47.8%)	35 (52.2%)	
Pathological gambling	4 (0.4%)	3 (75.0%)	1 (25.0%)	
Third diagnosed addictive disorder				0.0865
Patients with no third addiction diagnosis	709 (85.2%)	459 (64.7%)	250 (35.3%)	
Alcohol	8 (0.9%)	3 (37.5%)	5 (62.5%)	
Opioid	10 (1.2%)	5 (50.0%)	5 (50.0%)	
Opioid substitution	4 (0.5%)	3 (75.0%)	1 (25.0%)	
Cannabis	9 (1.0%)	3 (33.3%)	6 (66.7%)	
Sedatives/hypnotics	0 (0%)	0 (0%)	0 (0%)	
Cocaine	13 (1.5%)	10 (76.9%)	3 (23.1%)	
Multiple drug use	75 (9.0%)	39 (52.0%)	36 (48.0%)	
Pathological gambling	4 (0.5%)	3 (75.0%)	1 (25.0%)	
Other diagnosed disorders				
Yes	338 (40.7%)	246 (72.8%)	92 (27.2%)	**0.0001**
No	493 (59.3%)	278 (56.4%)	215 (43.6%)	

**Table 4b:** (b) Results of logistic regression analysis from medical history.

Characteristic	OR	95% CI	Wald Chi2	*p*
Addiction related comorbidity: infection				
Yes	1.00			
No	0.67	0.38–1.19	1.86	0.1732
Addiction related comorbidity: gastrointestinal disorder				
Yes	1.00			
No	0.88	0.63–1.25	0.48	0.4889
Addiction related comorbidity: CNS disorder				
Yes	1.00			
No	1.29	0.68–2.47	0.60	0.4379
Addiction related comorbidity: peripheral nervous system disorder				
Yes	1.00			
No	0.52	0.23–1.18	2.48	0.1153
First diagnosed addictive disorder				
Alcohol	1.00			
Opioid	**3.23**	**1.98–5.24**	**11.14**	**0.0008**
Other	**2.12**	**1.40–3.21**	**0.63**	**0.4282**
Second diagnosed addictive disorder				
Yes	1.00			
No	1.15	0.79–1.69	0.53	0.4668
Third diagnosed addictive disorder				
Yes	1.00			
No	1.02	0.62–1.67	0.01	0.9542
Other diagnosed disorders				
Yes	1.00			
No	**2.07**	**1.50–2.86**	**19.58**	**<0.0001**
